# Rrp15 affects cell cycle, proliferation, and apoptosis in NIH3T3 cells

**DOI:** 10.1002/2211-5463.12128

**Published:** 2016-10-06

**Authors:** Tao Wu, Mei‐Xia Ren, Guo‐ping Chen, Zheng‐ming Jin, Gang Wang

**Affiliations:** ^1^ Department of CardiologyThe First Affiliated HospitalSchool of MedicineZhejiang UniversityHangzhouChina; ^2^ Department of EndocrinologyThe First Affiliated HospitalSchool of MedicineZhejiang UniversityHangzhouChina; ^3^ Cancer Institute of Integrative MedicineTongde Hospital of Zhejiang ProvinceZhejiang Provincial Academy of Traditional Chinese MedicineHangzhouChina

**Keywords:** cell proliferation and apoptosis, NIH3T3 cell, RNA binding, Rrp15 gene

## Abstract

Riken 2810430M08 (hereinafter referred to as Rrp15) is a newly identified and reported gene from the mouse genome. In our previous work, we found that the gene had a relationship with the proliferation and activation of T cells. Rrp15 protein is highly homologous with RRP15 (budding yeast), which has an important role in ribosomal RNA processing. We explored the potential function of Rrp15 in apoptosis, cell proliferation, and its involvement with RNA in the nucleus. We constructed a knockdown of the Rrp15 gene in NIH3T3 cells and then performed real‐time PCR, western blotting, flow cytometry, and immunofluorescence to determine the function of the Rrp15 gene. Knockdown of the Rrp15 gene suppresses proliferation and induces apoptosis. We also found that the Rrp15 protein was normally distributed in the nucleus and bound to RNA or pre‐RNA in the nucleus. Additionally, Rrp15 altered the activity of the 20S proteasome. Rrp15 promotes proliferation and inhibits apoptosis in NIH3T3 cells and may have a relationship with RNA in the nucleus.

**Abbreviations**
DAPI: 4′,6‐diamidino‐2‐phenylindole
OD: optical density
PVDF: polyvinylidene fluoride
RIPA: radio immunoprecipitation assay
RRP15p: ribosomal RNA processing 15 homolog
Rrp15: Riken 2810430M08

The Riken 2810430M08 (hereinafter referred to as Rrp15) gene was first reported in the journal *Nature* by the Riken mouse gene Encyclopedia Project in 2001. The gene has little sequence homology with other mouse genes, and the Rrp15 protein has no inhibited functional domain 1. The Rrp15 gene is 700 kilobases and is localized on chromosome1 h5, and its 1228‐bp cDNA encodes for a protein consisting of 281 amino acids 1, 2.

Using a gene chip array ‘Mouse 15K cloned gene set, National Institute of Aging’, we found that expression of the Rrp15 gene had increased in activated T cells of C57BL/6 mice stimulated by antigens for 24 h. The peak expression of the gene first appeared at 5 h after stimulation and lasted for more than 24 h, as examined using Northern blotting. *In situ* hybridization revealed that the Rrp15 gene was closely correlated with the development of the mouse embryo 2.

The Rrp15 protein has a region consisting of 92–216 amino acid residues that are highly homologous to the budding yeast, RRP15 (ribosomal RNA processing 15); this region is called ribosomal RNA processing 15 homolog (RRP15p). RRP15p was identified in 2005 and is involved in ribosomal RNA processing, particularly the processing of pre‐rRNA. Pre‐rRNA processing is a procedure in which rRNA is transcribed from genomic rDNA during endo‐ or exonuclease digestion and methylation by regulating proteins before it becomes mature rRNA 3. This is a key preparatory step in the assembly of large and small subunits of ribosomes 3. Several reports have indicated that ribosomal processing proteins are highly evolutionarily conserved in eukaryotes 3, 4, 5, 6, 7, 8. In higher level organisms, there are many proteins that demonstrate homology to the RNA processing factors of yeast, for example, Nop25 5, Nop52, 6 and Bop1^7^. Therefore, the Rrp15 gene in mice may have similar conserved functions on mature rRNA as RRP15p in yeast. In eukaryotic cells, including T cells, dysfunction of mature of rRNA impacts the growth of cells 3, 4, 5, 6, 7, 8, 9, 10, 11, 12. When rRNA processing proteins such as RRP15 inhibit cell growth and proliferation, cells also exhibit suppressed function of mature ribosomal subunits 3, 11, 12.

Our preliminary work showed that the correlation between Rrp15 with the activation and proliferation of T cells is consistent with the functional characteristics of rRNA processing proteins. Levels of Rrp15 and other rRNA processing proteins are higher when activation of T cells is stimulated with antigens in order to maintain the required protein necessary for cells in the proliferation phase. Therefore, we speculated that Rrp15 and RRP15p have a similar function.

## Materials and methods

### Cell culture

Studies were conducted in a murine embryonic fibroblast cell line (NIH3T3), which was purchased from Shanghai National Type Collection Center and was cultured in Dulbecco's Modified Eagle's Medium (DMEM, Jinuo, Hangzhou, China) supplemented with 10% (v/v) FBS (Hyclone, Logan, UT, USA) and 1% penicillin/streptomycin 13.

### Rrp15 gene knockdown shRNA lentiviral vector construction

The ORF of Rrp15 gene (Rrp15 mouse) (NM_026041.2) was cloned from mouse cells used in the construction of the overexpression vector. Lentiviral overexpression vectors (Lent‐Rrp15), the knockdown (Lent‐Rrp15i) of the mouse Rrp15 gene, and control vectors (Lent‐Con) were purchased from GenePharma (Shanghai, China). Lentivirus infection was performed according to the manufacturer's instructions (http://www.genepharma.com).

### Cell proliferation assay

NIH3T3 cells were seeded in 96‐well plates at 5000 cells per well with 100 μL of medium. After 24, 48, and 72 h, 10 μL of CCK‐8 (Beyotime, Jiangsu, China) was added to each well and incubated at 37 °C for 2 h before measuring the absorbance at 450 nm using a spectrometer (Bio‐Rad, Hercules, CA, USA).

### Flow cytometry for apoptosis and cell cycle assay

NIH3T3 cells with overexpressed or down‐regulated Rrp15 gene expression were processed for analysis of apoptosis and cell cycle using flow cytometry. Annexin V‐PE was performed for detection of preapoptosis, and 7‐AAD was used for nuclear staining. To detect apoptosis, cells (5 × 10^4^) were planted in a 24‐well plate and after a period of incubation, cells were harvested by incubation with Annexin‐V and propidium iodide (PI) solution. All cells were analyzed by flow cytometry. For cell cycle analysis, cells were harvested and fixed in 70% (v/v) ethanol and then wash in PBS. All cells were stained with 7‐AAD solution, and the DNA content of each cell was detected by flow cytometry 14.

### Immunofluorescence staining and subcellular localization

NIH3T3 cells in six‐well plates were washed twice with cold PBS and then fixed with methanol/acetone (v : v = 1 : 1) followed by washing with PBS. After blocking in 1% BSA for 1 h at room temperature, cells were incubated with a mouse anti‐tubulin antibody (Abcam, Cambridge, MA, USA) for 1 h. Following washing with PBS, cells were incubated with Alexa Fluor 594‐conjugated secondary antibody followed by 4′,6‐diamidino‐2‐phenylindole (DAPI) stain (Life Technologies, Foster City, CA, USA). Finally, cells were imaged using a laser scanning fluorescence microscope (Olympus, Tokyo, Japan) 15.

### 20S proteasome activity assay

A Proteasome 20S Activity Assay Kit (Abcam) was used for the 20S proteasome activity assay. Briefly, approximately 2 × 10^6^ cells were centrifuged, and the pellet was suspended in 0.5% NP‐40 solution followed by centrifuging to remove insoluble matter. The OD value was quantified using a spectrometer 16.

### Western blot analysis

Cells were lysed in RIPA buffer, followed by the determination of protein concentration and denaturation of total proteins. SDS/PAGE was performed for protein separation, followed by transfer to a polyvinylidene fluoride (PVDF) membrane (Millipore, Bedford, MA, USA). The blots were incubated with primary and secondary antibodies, followed by staining and quantification with an imaging system (Bio‐Rad) 16.

### Real‐time quantitative PCR

Total RNA of the NIH3T3 cells was extracted with TRIzol (Life Technologies) according to the manufacturer's instructions. After cDNA was synthesized by reverse transcription of 500 ng of total RNA with a PrimeScript^™^ RT reagent Kit with gDNA Eraser (Perfect Real Time) (TAKARA, Dalian, China), quantitative RT‐PCR was performed using StepOne Plus (AB, USA) with tSYBR^®^ Premix Ex Taq^™^ (TliRNaseH Plus), ROX plus (TAKARA). The RT‐PCR primer for the Rrp15 gene and internal control gene actin were purchased from GenePharma. The PCR protocol used was 95 °C for 5 min, 40 cycles at 95 °C for 10 s, 60 °C for 34 s followed by a melting curve step of 60 °C up to 95 °C. The relative expression of the Rrp15 gene was normalized to the control gene actin 17.

## Results

### The Rrp15 gene impacts proliferation, cell cycle, and apoptosis of NIH3T3 cells

**Figure 1:**
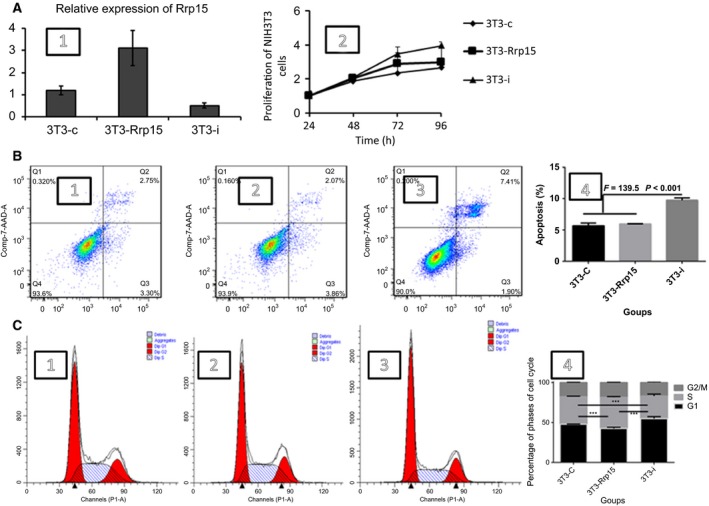
Knockdown of Rrp15 reduced proliferation, increased apoptosis, and blocked cell cycle of the NIH3T3 cells. (A2) Proliferation of the NIT3T3 cells, (B) apoptosis of the NIH3T3 cells detected using flow cytometry and 3T3‐i group has a higher apoptosis than other groups (*P* < 0.01, ***) (B4), and (C) cell cycle of the NIH3T3 cells examined using flow cytometry. S phase of 3T3‐Rrp15 cells has a more percentage than other groups (*P* < 0.001, ***), that means Rrp15 could promote cell cycle (C4).

Previously, we have found that the Rrp15 gene is expressed in the permanent cells of the fetal mouse, such as neurons 2. Despite that, we aimed to determine the role of the Rrp15 gene in other types of tissues or cells. We transfected NIH3T3 cells with lentiviral vector harboring Rrp15 gene and constructed stable Rrp15‐expressing cells (3T3‐Rrp15) with lentiviral vectors. Additionally, control cells (3T3‐c) and knockdown cells (3T3‐i) were constructed. Expression of the Rrp15 gene was assessed using RT‐PCR, and we found that expression of the Rrp15 gene was increased by threefold in 3T3‐Rrp15 cells and decreased by twofold in 3T3‐i cells compared to control cells (3T3‐c) (Fig. 1A1). To determine the effects of the Rrp15 gene on proliferation, cell cycle, and apoptosis, we performed a CCK‐8 assay and flow cytometry. The CCK‐8 assay showed that 3T3‐Rrp15 cells proliferated significantly faster compared to all other cells (Fig. 1A2).

Previous reports indicated that deletion of Rrp15p (the homologous Rrp15 gene in humans) resulted in an induction of cell cycle arrest and expression of p21, a cell cycle inhibitor 18. Therefore, flow cytometry was carried out to determine the role of the Rrp15 gene on cell cycle and apoptosis in the NIH3T3 cells. We found differences in apoptosis rates and cell cycle among 3T3‐Rrp15, 3T3‐i, and 3T3‐cells (Fig. 1B, C). As shown in Fig. 1, cells overexpressing the Rrp15 gene and control cells both showed a decrease in the percentage of apoptosis, and knockdown of the Rrp15 gene may inhibit cell cycle at the G1/S phase check point (Fig. 1C). Therefore, the Rrp15 gene may have an important role in cell cycle and apoptosis in the fibroblast NIH3T3 cells.

### The Rrp15 gene promotes the cell cycle process through p21, p53, Cyclin D1, and Cyclin A

**Figure 2:**
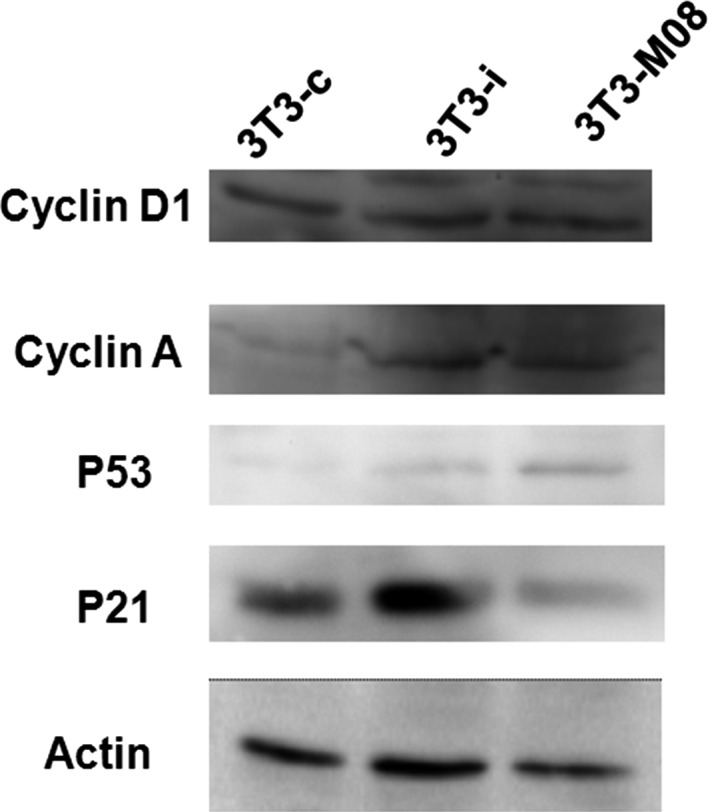
Western blot analysis of cell cycle‐associated proteins. Overexpression or down‐regulation of the Rrp15 gene (3T3‐Rrp15 and 3T3‐i, respectively) impacts the protein levels of Cyclin D1, Cyclin A, p53, and p21 compared to the control group (3T3‐c).

After confirmation that cell cycle was inhibited by knockdown of the Rrp15 gene, we performed RT‐PCR and western blotting to detect genes associated with the cell cycle. P53, an anticancer gene, controls cell DNA damage by inducing p21 expression to block cell cycle arrest at the G1 phase. The Cyclin proteins, such as Cyclin D1 and Cyclin A, bind to Cyclin‐dependent kinases to allow transition to the next phase of the cell cycle; Cyclin D1 and Cyclin A are essential to control the G1/S transition 19. We examined the expression of p21, p53, Cyclin D1, and Cyclin A in the 3T3‐Rrp15, 3T3‐i, and 3T3‐c cells (Fig. 2). We found that the cell cycle‐promoting genes, Cyclin D1 and Cyclin A, were down‐regulated in cells overexpressing the Rrp15 gene. Conversely, we found that expression of P53 and p21 were up‐regulated in the 3T3‐Rrp15 cells. These data suggest that the Rrp15 gene is involved in regulation of cell cycle proteins either directly or indirectly.

### Subcellular distribution of the Rrp15 protein in NIH3T3 cells

**Figure 3:**
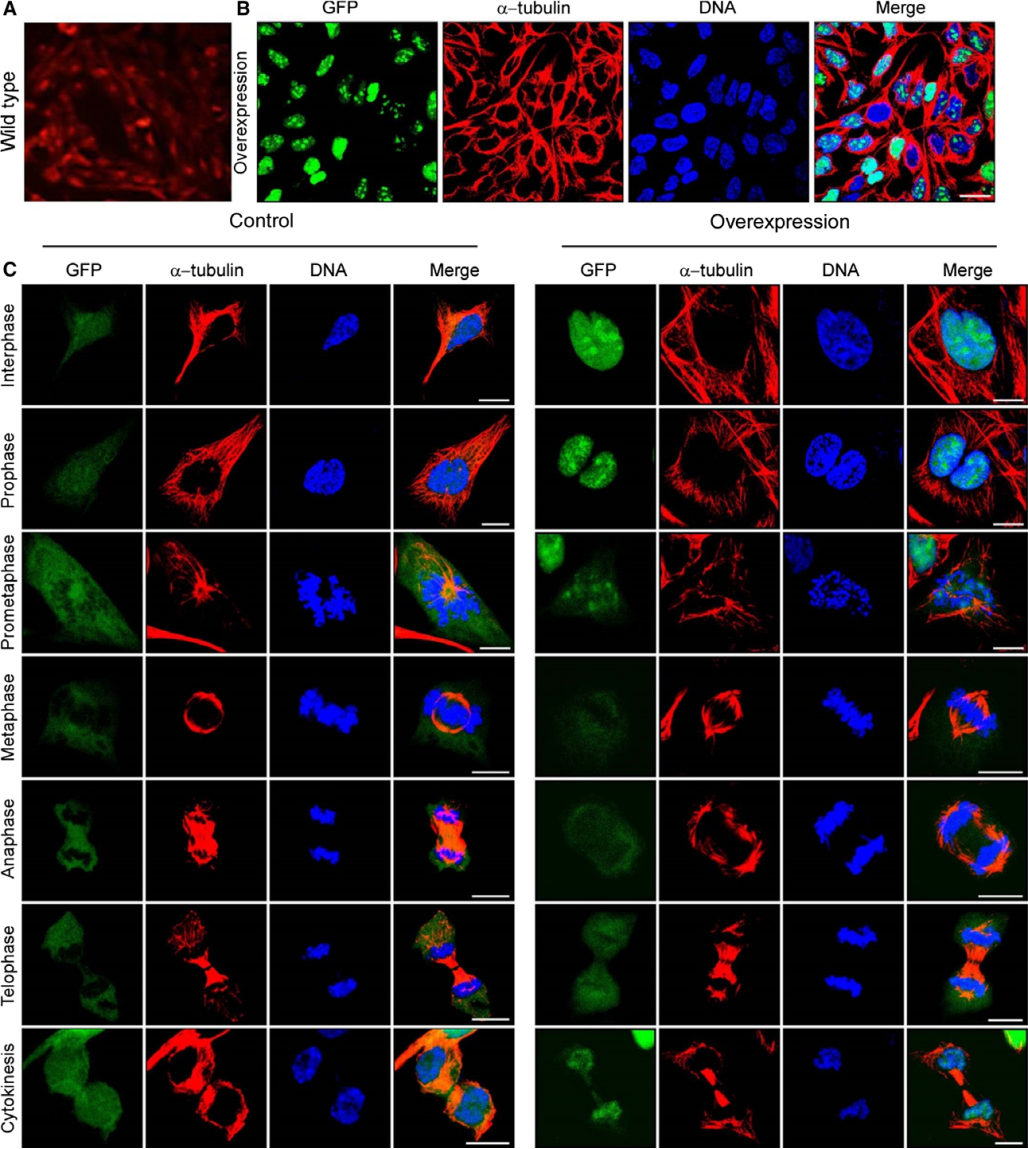
Subcellular localization of the Rrp15 protein in quiescent and cell‐cycling NIH3T3 cells. (A) Expression of the Rrp15 gene in wild‐type cells, (B) GFP‐Rrp15 fused protein in quiescent cells (GFP stands for Rrp15 protein), and (C) in control and overexpressing cells, subcellular localization of the Rrp15 protein in cells during the different stages of cell cycle (GFP stands for Rrp15 protein).

We used immunoflouresence to determine the subcellular distribution of the Rrp15 protein in NIH3T3 cells. We determined that the Rrp15 protein was localized in the cell nucleus (Fig. 3A, red). Confocal immunofluorescence imaging showed that the Rrp15 protein was mainly in the nucleus of 3T3‐Rrp15 cells (Fig. 3B, green), and theRrp15 protein was colocalized with chromosomes during the gap phase, prophase, prometaphase, and cytophasmic division, but not during metaphase, anaphase, or telophase (Fig. 3C). Additionally, there was a decrease in colocalization during the transition from prophase into prometaphase (Fig. 3C). These data suggest that the Rrp15 protein functions in the nucleus and is involved in mitogenesis by binding to the chromosome. Furthermore, the subcellular location of the Rrp15 protein suggests its involvement in the processing of mature and precursor rRNA.

### The Rrp15 protein is relative to rRNA in the nucleus

**Figure 4:**
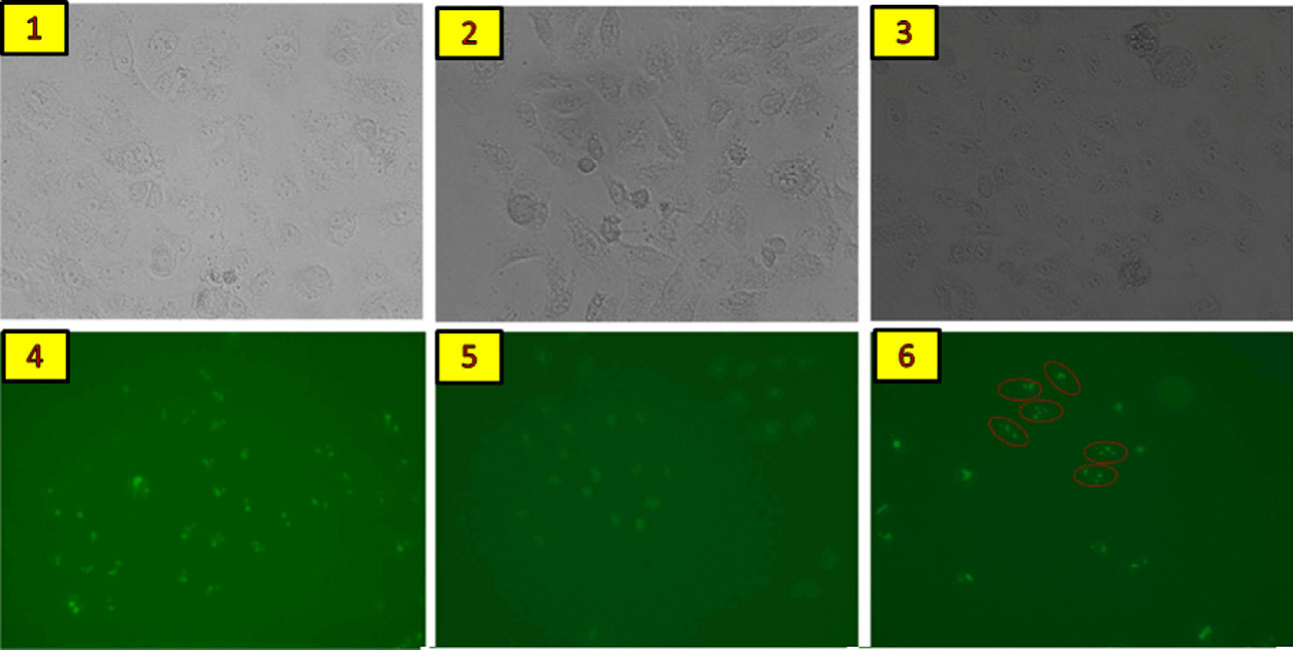
The Rrp15 protein closely bound RNA in NIH3T3 cells. 1 and 4 were control cells, 2 and 5 were cells with the Rrp15 gene knocked down, and 3 and 6 were cells that overexpressed theRrp15 gene. In Figure F, red circles indicate loosening and scattering of the Rrp15 protein after RNase digestion.

It has been reported that Rrp15p, a homologous protein of the Rrp15 protein, is involved in the synthesis of large subunit rRNA 4, 20. To determine if the Rrp15 protein has a similar function, GFP‐labeled NIH3T3 cells overexpressing Rrp15 were fixed with or without RNase digestion. We found the Rrp15 protein was bound together unusually tight (Fig. 4) and loosened after adding the RNase (Figure, Green dot). The data indicate that Rrp15, like Rrp15p, may have an important role in the functions of RNA and perhaps rRNA at the area of cellular nucleus.

### The activity of the 20S proteasome is influenced by the Rrp15 protein

**Figure 5:**
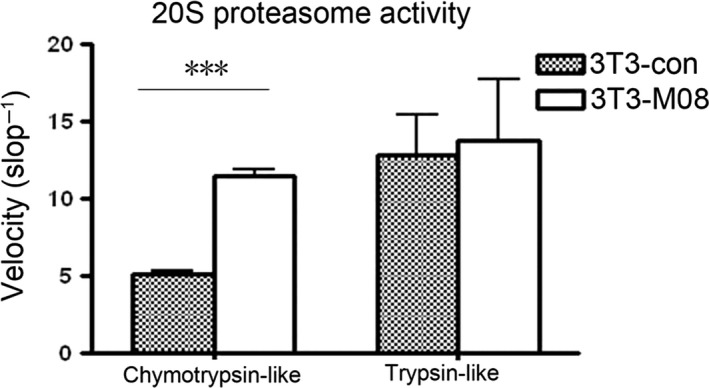
The Rrp15 gene has an impact on the 20S proteasome chymostrypsin‐like and trypsin‐like activity. Overexpression of the Rrp15 gene significantly increased chymostrypsin‐like activity (*P* < 0.01, ***), but not trypsin‐like activity, of the 20S proteasome.

Proteasomes are found in all eukaryotic cells and consist of two subunits, 20S and 60S, which are responsible for degrading proteins with specific tags. The 20S subunit is a protease with chymotrypsin‐like, trypsin‐like, and caspase‐like protease activities. Ribosome proteins crosstalk with proteasomes. The chymotrpsin‐like activity of the 20S proteasome increased significantly with the increased levels of the Rrp15 protein compared to the normal Rrp15 levels (*P* < 0.001). However, there was no difference in trypsin‐like activity between the two NIH3T3 cells. Chymotrypsin‐like activity of the 20S proteasome was selectively influenced by the Rrp15 protein suggesting the Rrp15 protein affects the core subunit of the 20S (Fig. 5).

## Discussion

In the present work, we found that the Rrp15 gene is involved in the promotion of cell proliferation and cell cycle and in the inhibition of apoptosis. The Rrp15 protein is distributed in the nucleus of cells and has potential roles involving the processes of RNA, which may include mRNA and rRNA, and the activity of the 20S proteasome in cells. This is the first report suggesting a role of theRrp15 protein in RNA‐associated processes and 20S proteasome activity.

In terms of amino acid residues, the Rrp15 protein is highly homologous to the budding yeast RRP15p protein, which may have a relationship with the procedures of nuclear RNA, particularly pre‐rRNA 3. In our experiment, the Rrp15 protein was confirmed to have a similar role to the RRP15p protein. We found the Rrp15 protein was bound with RNA in the nucleus but dissociated after adding RNase. This allowed RNase to digest the RNA, including pre‐RNA, mature RNA, rRNA, and microRNA, into smaller fragments. Proteins participating in RNA processing are removed following RNase digestion in the nucleus 4. The process of ribosome assembly is evolutionally conserved 4, 21. RRP15p is a component of the pre‐60S ribosome in the nucleus and depletion of the RRP15p gene inhibits the accumulation of several pre‐rRNA of the 60S ribosome 4. The function of the Rrp15 protein in pre‐rRNA maturation should be investigated in future studies. Ribosome‐associated proteins involved in the maturation of ribosomes, such as Rrp14 and Rrp12, generally function in regulating proliferation, apoptosis, and cell cycle 22.

The Rrp15 gene also inhibits proteasomes. Inhibition of proteasome may lead to the induction of apoptosis and thereby block cell growth 23, 24. Proteasome inhibitors, bortezomib and YSY01A, also target apoptosis and cell cycle 24, 25. We showed that the Rrp15 gene also has effects on cell cycle and apoptosis. The 20S proteasome is a 700‐kDa complex unit, consisting of 28 proteins 26. Proteasome inhibition has been shown to have anticancer effects due to hyperactivation of proteasomes in cancer cells 27. The role of the Rrp15 gene in cell cycle and apoptosis may be associated with the inhibition of the 20S proteasome and should be investigated further.

In this study, we are the first to describe the role of the Rrp15 gene in proliferation, apoptosis, and the activity of proteasomes. Further investigations of the Rrp15 gene are necessary to fully elucidate the functions of this gene.

## Conclusions

In our work, the Rrp15 protein was normally distributed in the nucleus and bound to RNA or pre‐RNA in the nucleus. Meanwhile, Rrp15 could alter the activity of the 20S proteasome, promote proliferation, and inhibit apoptosis in NIH3T3 cells. Additionally, Rrp‐15 might have a relationship with RNA in the nucleus, especially ribosomal RNA.

## Author contributions

TW designed research; TW, MR, GC, and ZJ performed research; TW analyzed data; and TW and GW wrote the paper.
